# Physicochemical cell disruption of *Bacillus* sp. for recovery of polyhydroxyalkanoates: future bioplastic for sustainability

**DOI:** 10.1007/s13205-024-03913-y

**Published:** 2024-02-02

**Authors:** G. Sohani Bhat, B. K. Deekshitha, V. Thivaharan, M. S. Divyashree

**Affiliations:** Department of Biotechnology, Manipal Institute of Technology, Manipal Academy of Higher Education, Manipal, 576104 India

**Keywords:** Polyhydroxyalkanoates, Bioplastic, Intracellular, Biocompatible, Downstream process, Sustainable

## Abstract

Polyhydroxybutyrate (PHB) is known for wide applications, biocompatibility, and degradability; however, it cannot be commercialized due to conventional recovery using solvents. The present study employed mechanical cell-disruption methods, such as Pestle and mortar, sonication, and glass bead vortexing, for solvent-free extraction of PHA from *Bacillus* sp. Different time intervals were set for grinding (5, 10, 15 min), sonicating (1, 3 and 5 min), and vortexing (2, 5 and 8 g glass beads with 5, 10 and 15 min each) hence studying their effect on cell lysis to release PHA. Tris buffer containing phenylmethyl sulfonyl fluoride (PMSF) (20 mM Tris–HCl, pH 8.0, 1 mM PMSF) was employed as a lysis buffer to study its action over *Bacillus* cells. Its presence was checked with the above methods in cell lysis. Sonicating cells for 5 min in the presence of lysis buffer achieved a maximum PHA yield of 45%. Cell lysis using lysis buffer yielded 35% PHA when vortexing with 5 g glass beads for 15 min. Grinding cells for 15 min showed a maximum yield of 34% but lacked a lysis buffer. The overall results indicated that the action of lysis buffer and physical extraction methods improved PHA yield by %. Therefore, the study sought to evaluate the feasibility of applying laboratory methods for cell disruption. These methods can showcase possible opportunities in large-scale applications. The polymer yield results were compared with standard sodium hypochlorite extraction. Confirmation of obtained polymers as polyhydroxy butyrate (PHB) was made through FTIR and ^1^HNMR characterization.

## Introduction

Although multifaceted waste management solutions are undertaken, conventional plastics pollute various ecosystems. These plastics have driven greater significance globally with wide applications and high durability. Microplastics are a major pollutant concern, specifically in aquatic systems. Weathering and wearing larger conventional plastics results in smaller microplastics reaching the environment (Cverenkárová et al. 2021; Zhou et al. 2023). Studies on exposure and presence of nano- and microplastics within human tissues and body systems are emerging scientific topics (Cverenkárová et al. 2021; Ragusa et al. 2021). Biodegradable polymers are, thus, gaining forefront interest as potential substitutes for conventional plastics. With higher degradability, biocompatibility, water-insoluble, optically active, isotactic, and piezoelectric, and being obtained from cheaper carbon sources, polyhydroxyalkanoates (PHAs) are known to be the most renowned biopolymer (Yukesh Kannah et al. 2022). PHA shows properties like several conventional plastics such as polyethylene (PE), polypropylene (PP), polyvinylchloride (PVC), and polyethylene terephthalate (PET) (Cverenkárová et al. 2021; Saavedra del Oso et al. 2021; Pooja et al. 2023).

Production of PHA varies among various bacteria, wherein about 90 genera, including 300 species, have been reported in the production of PHA. Bacterial isolates are reported to be isolated from diverse habitats. Species of *Pseudomonas*, *Bacillus* is observed to be a highly reported genus in the production of PHA (Mascarenhas and Aruna 2017; Mohapatra et al. 2017; Kosseva and Rusbandi 2018; Mohammed et al. 2019). PHAs are intracellular, organic, water-insoluble, nano-sized, granular cytoplasmic inclusions, ranging in size from 0.2 to 3 µm, with a polyester core surrounded by phospholipids (Poli et al. 2011). Considering chain length, it has been categorized as short-chain length (scl), medium-chain length (mcl), and long-chain length (lcl) PHAs with 3–5, 6–14, and 15 and above monomeric building blocks of carbons, respectively. Scl-PHAs (congeners of 3-hydroxybutyrate and 3-hydroxy valerate) and mcl-PHAs (congeners of 3-hydroxyhexanoate, 3-hydroxyoctanoate, and 3-hydroxydecanoate) are most known. Meanwhile, lcl-PHA is rarely known and less studied (Muneer et al. 2020; Sharma et al. 2021; Zhou et al. 2023). Poly-3-hydroxybutyrate (PHB), a scl-PHA, is the most studied member of the PHA family. It was reported to be the first discovered PHA in 1926 by Maurice Lemoigne as a *Bacillus megaterium* component (Kumar et al. 2020). PHB shows similar isotactic properties to polypropylene. It is also known to have diverse applications, specifically medical, due to high porosities observed under the Scanning electron microscope (SEM) (Bergstrand et al. 2014).

The chemical digestion method using solvents is the renowned way of extraction. Due to non-eco-friendly and cost-intensive solvent extraction methods, there is limited production of PHAs. Academia and industries are developing advanced ‘green methods’ of PHAs recovery (Daly et al. 2018; Prasad and Sharma 2019). Extraction methods known to date can be categorized into physical, chemical, and biological methods. These methods need to be scrutinized as a possible green extraction process. Discussion on extraction methods reported to date and their pros and cons are available in the literature (Kunasundari and Sudesh 2011; Shahid et al. 2021). Green extraction methods are known to involve mild chemicals or devoid of hazardous chemicals and methods during recovery. Non-halogenated solvents, ionic-liquid, deep eutectic solvents, and green solvents are evolving as environmentally friendly processes of recovery of biopolymers like PHA (Hecht et al. 2010; Dubey et al. 2017; Prasad and Sharma 2019).

Hence, the current study investigated the combination of physical and chemical modes of PHB extraction. Glass beads were involved in the lysis of *Bacillus* cells, and recovery of intracellular PHB was studied for the first time. Nevertheless, a similar approach or pre-treatment during cell lysis was investigated to recover various biomolecules and PHA, respectively (Ramanan et al. 2008; Samorì et al. 2015). This laboratory technique can be compared to the vertical bead milling technique for extensive testing.

Sonication is one of the age-old methods employed in the cellular disruption of diverse cell lines. Ultrasound irradiation is being extensively studied as a method of bacterial cell lysis, making the extraction system an efficient mass transfer for releasing PHA. Operation time and energy of ultrasonication are the key parameters to be cleared before operations (Zou et al. 2023). Besides this, the nature of cells to be disrupted must be considered. From the literature, sonication was notably used as a pre-treatment step, assisting other extraction methods instead as a sole method.

In addition, grinding cells as a part of the abrasive force, using Pestle and mortar, is another traditional method of cell disruption. Although this method is specifically used in plant cells, studies are flourishing by experimenting on broader cell lines, including microbes (Harrison 1991; Nemer et al. 2021). Grinding bacterial cell mass using a Pestle and mortar was studied in the present study. As a combination, the effect of lysis buffer assisting the above methods in cell lysis was also studied. The present approach has made the study part of the ‘green method’ of extraction, devoid of lysis buffer. Therefore, it contributes to the investigation of new opportunities for sustainability. The polymer obtained was characterized using FTIR, ^1^H NMR, and G.C.

## Materials and methods

### Materials and media preparation

All the reagents and chemicals required to prepare nutrient agar plates, nutrient broth, lysis buffer, and PHA production media were procured from Loba Chemie (India). The experiment’s 0.5 mm diameter of glass beads was procured from Himedia (India).

20 mL of nutrient broth was prepared in 50 mL of Erlenmeyer flask. 200 mL of PHA production media was prepared in a 1 L Erlenmeyer flask. Production media included (g/L): Na_2_HPO_4_ 2H_2_O 2.2, (NH_4_)_2_SO_4_ 1.5, KH_2_PO_4_ 1.5, MgSO_4_·7H_2_O 0.2, Sucrose 20. The pH of the media was set to 7.0 using a pH meter (Eutech Instruments, India) (Geethu et al. 2019). Lysis buffer composition was taken in the literature (Ramanan et al. 2008). Tris buffer containing phenylmethyl sulfonyl fluoride (PMSF) (20 mM Tris–HCl, pH 8.0, 1 mM PMSF) was prepared to 100 mL.

### Culture maintenance and inoculum preparation

*Bacillus* sp. isolated from the soil sample was sub-cultured on a sterile nutrient agar plate and maintained at 4 °C for further experimentation. Inoculum was prepared by taking 2–3 loopful of culture inoculated into sterile 20 mL nutrient broth and incubated overnight at 32 °C under shaking at 180 rpm. The inoculum contained 2.1 × 10^–8^ CFU/ml cells.

### Characterization of isolate: microscopy

#### Gram staining, spore staining, and scanning electron microscope

*Bacillus* smear was prepared, followed by heat fixing. Heat-fixed cells were subjected to Gram staining (Smith and Hussey 2005), endospore staining according to (Hussey and Zayaitz 2007), and scanning electron microscopy (SEM) (Kaláb et al. 2008) were performed. SEM observations (Carl Zeiss, Germany) were carried out for bacterial isolate, and images were captured at 10.00 K× at 10 kV.

### Screening of PHA-producing isolate

#### Sudan black B

The isolates of bacteria obtained were screened for PHA-positive tests using Sudan black B (SBB) as a preliminary test to detect the presence of intracellular PHA granules. Isolates were stained with SBB per the literature’s staining protocol (Kavitha et al. 2016).

#### Nile blue

This method aids in being more specific, rapid, and highly efficient. The isolates showing a positive response for SBB were screened for confirmation with Nile blue A staining (Spiekermann et al. 1999).

### PHA production and extraction

#### Inoculation and PHA production

*Bacillus* isolate as an inoculant was inoculated in nutrient broth media, and the overnight culture grown at 30 °C was transferred in its log phase into PHA production media (PM) composition (g/L): Na_2_HPO_4_. 2H_2_O 2.2, KH_2_PO4 1.5, (NH4)_2_SO_4_ 1.5, MgSO_4_7H_2_O 0.2, Sucrose 20. 200 mL of PM was taken in 1 L Erlenmeyer flask. The inoculum transferred was 10%. The culture broth was incubated in a shaker for 72 h at 30 °C for 180 rpm. Post-incubation, a culture broth of 50 mL each was processed for extraction. The cell pellet was obtained by centrifugation of culture broth for 5 min at 8430×g. The cell’s dry weight was obtained by drying the biomass at 80 °C to constant dry weight. This known weight was used to estimate the weight of wet biomass for all extraction methods.

### PHA extraction by a conventional method

The dry cells were hydrolyzed with 4% sodium hypochlorite incubated at 37 °C for 1 h. After digestion of non-PHA cell mass (NPCM), the PHA was washed with water and acetone. The PHA pellet was dissolved in chloroform and evaporated to constant weight.

### Sonication, grinding, and glass beads vortexing of *Bacillus* cells

Cell disruption using sonication and Pestle and mortar were studied separately at varied conditions. The literature shows that sonication time as long as 10 min and beyond was known to decline (Burden 2012; Ishak et al. 2016). In the present study, sonication was performed at 1, 3, and 5 min intervals with pulse maintained at 30 s on and 30 off. Biomass was sonicated, containing 10 mL of sterile distilled water. Likewise, with 10 mL water, using a Pestle and mortar, biomass was hand-ground for 5, 10, and 15 min.

For the glass bead vortexing method, biomass was vortexed with 0.5 mm diameter glass beads in varied ratios (2 g, 5 g, and 8 g), each containing 10 mL water, vortexed for a duration of 5, 10, and 15 min. A similar method was repeated with 10 mL distilled water in place of lysis buffer.

This experimental setup was a modified cell lysis method employed in protein release from *Escherichia coli* cells (Ramanan et al. 2008). Besides, glass beads were used as a mechanical pre-treatment step in PHA recovery from mixed microbial culture (Samorì et al. 2015).

All the above methods were performed using the 10 mL lysis buffer containing Tris buffer with phenylmethyl sulfonyl fluoride (PMSF) (20 mM Tris–HCl, pH 8.0, 1 mM PMSF) instead of water. PMSF is known to be used as a lysis buffer from the literature (Ramanan et al. 2008). In addition, the lysis buffer effect was studied by vortexing wet biomass with 10 mL lysis buffer devoid of glass beads.

**Fig. 1:**
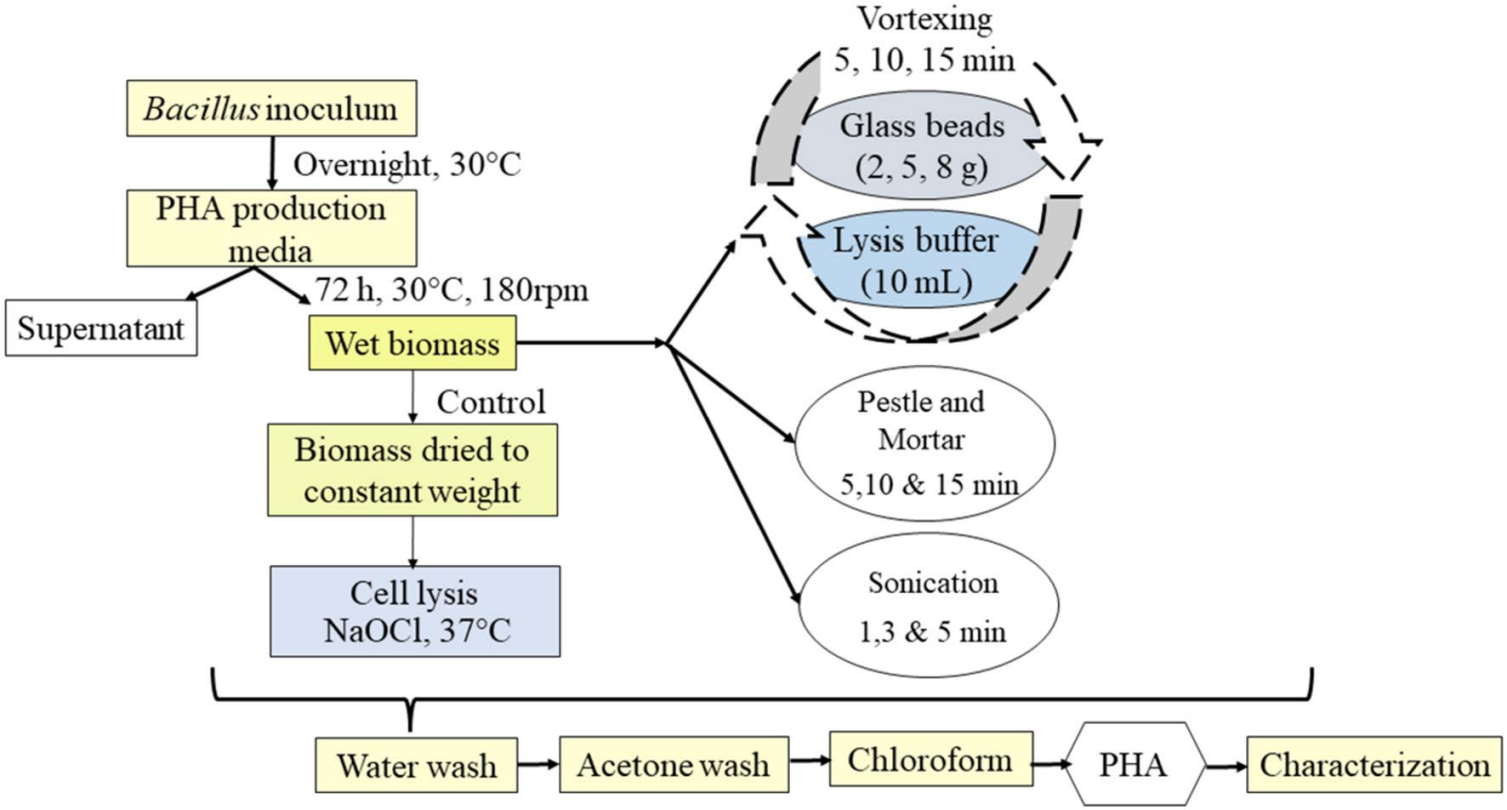
Schematic flowchart showing the PHA recovery from *Bacillus* sp. by conventional and physicochemical methods

After cell lysis, the lysate was centrifuged by washing with water successively with acetone and allowed to dry. The dried content was treated with chloroform by filtration and evaporated to dry weight. The PHA obtained was calculated in percentages based on dry weight (wt%). All the experiments were performed in duplicates. The schematic flow chart in Fig. 1 briefly explains the PHA extraction process using the standard solvent method as the control and physicochemical methods studied.

### Washing and reusing glass beads

After experimenting, the glass beads used were decontaminated using an autoclave. It was rinsed and washed with water and a laboline chemical. After thoroughly washing with water, beads were sterilized by dry heat using a hot air oven set at 120 °C for 30 min. Hence, it is stored for further use.

### Polymer characterization

The PHA sample recovered by physicochemical extraction was characterized by Fourier transform infrared Spectroscopy (FTIR), nuclear magnetic resonance (NMR), and differential scanning calorimetry analysis.

### Fourier transform infrared spectroscopy (FTIR)

Polymer film extracted from *Bacillus* sp. was analyzed spectroscopically by FTIR IRSpirit, QATR-S single reflection ATR accessory (Shimadzu, Japan). 4000 to 400 cm^−1^ range of infrared radiation was used in the analysis (Shamala et al. 2009). 4–5 mg of polymer sample was used in the analysis. FTIR analysis from the literature studies explored functional groups and various conformation bands in recovered PHA films.

### Nuclear magnetic resonance (NMR)

The recovered PHA films were subjected to ^1^HNMR analysis to elucidate the chemical structure. 4–5 mg of PHA samples were dissolved in 1 mL deuterated chloroform (CDCl_3_) (Salgaonkar et al. 2013). Spectra of NMR were recorded in Ascend 400 NMR spectrophotometer (Bruker, Germany).

### Differential scanning calorimetry (DSC)

#### Gas chromatography (G.C.)

After the extraction of PHA, its composition was analyzed by G.C. (Clarus 590, Perkin Elmer). 2 mg of dried biomass was subjected to methanolysis, and 1 mL chloroform, 0.8 mL methanol, and 0.15 mL of concentrated sulphuric acid were added and then heated at 80 °C for 2 h (Anil Kumar et al. 2007).

## Results and discussion

### Characterization of isolate

**Fig. 2:**
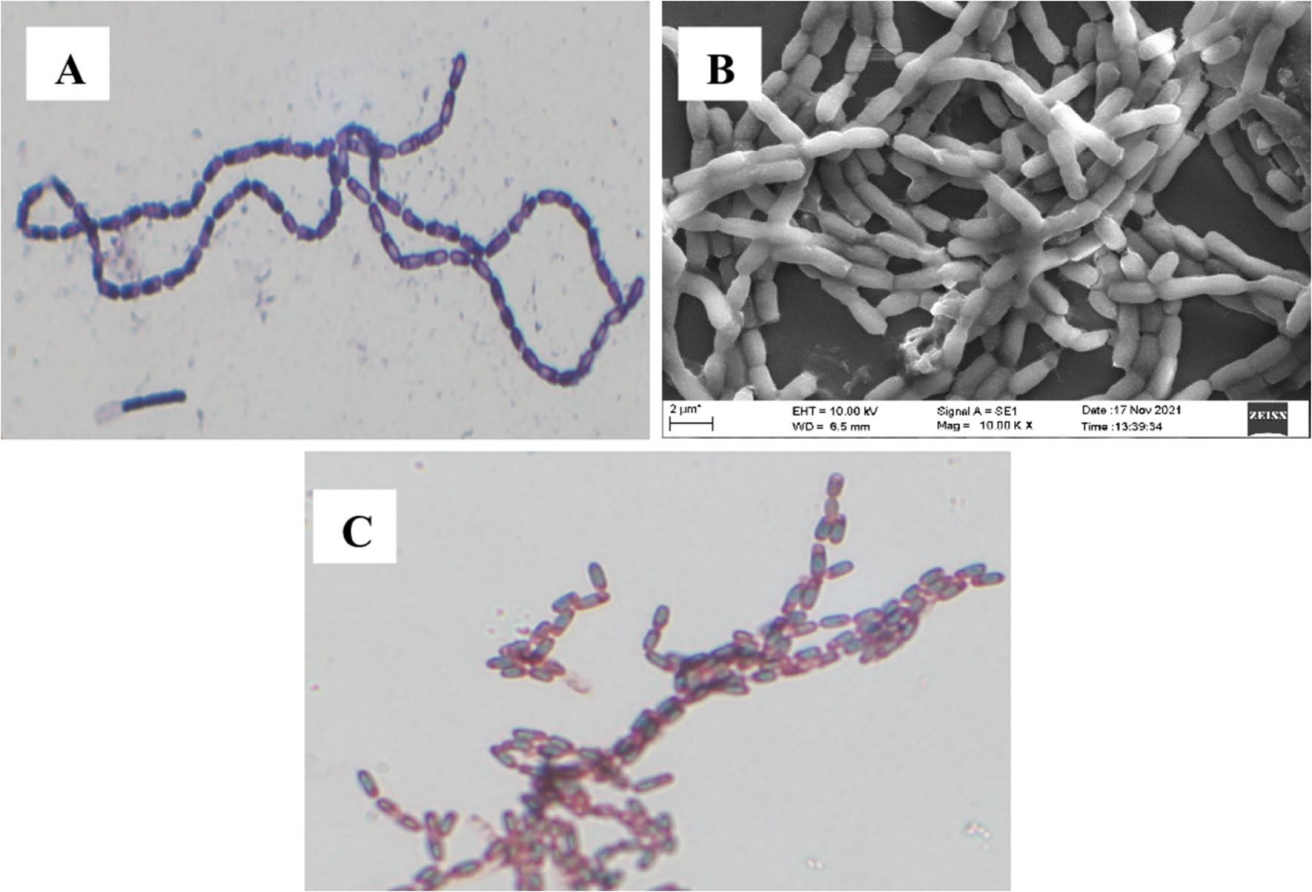
Characterization of bacterial isolate: **A** Gram staining showing rod-shaped cells stained for crystal violet observed under 100× oil immersion. **B** Rod-shaped cells observed under SEM at 10.00× magnification. **C** Endospore and vegetative cells stained in green and pink colors under 100× oil immersion

The screened isolate was observed as Gram-positive, staining purple color after Gram staining, as in Fig. 2A, under 100 X oil immersion. SEM confirmed further as rod-shaped chain cells with 10.00 K magnification as in Fig. 2B. Spore staining is shown in Fig. 2C. Stained spores are green in color within pink vegetative cells. Therefore, Gram and spore staining confirmed the isolate to be Gram-positive and endospore-producing *Bacillus* sp.

### Screening of PHA-producing isolate

**Fig. 3:**
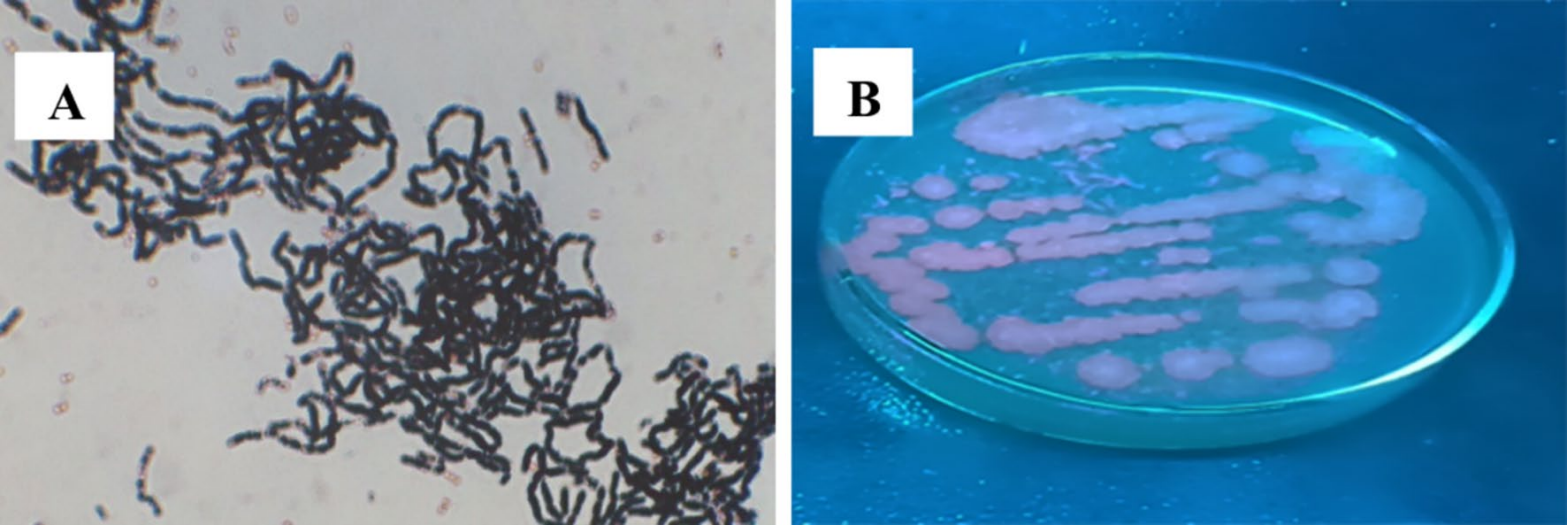
Screening PHA-producing isolate: **A** Sudan Black B staining of PHB granules under 100× oil immersion. **B** Fluorescence observed under U.V. showing positive for Nile red

Accumulation of intracellular PHA as black color granules was observed under a microscope in 100× oil immersion, as shown in Fig. 3A. Nile red staining, a fluorescent dye, being a specific PHA stain, showed fluorescence of PHA-producing isolates as in Fig. 3B, further confirming PHA’s presence. Since PHA granules are lipoidal, these granules can be visualized by lipid-soluble dye Sudan Black B that stains dark blue to black. Nile red staining distinguishes PHA accumulating and non-accumulating strains by staining PHA as bright orange (Aljuraifani et al. 2019; Chou et al. 2023; Jeevitha et al. 2023).

### PHA extraction using various physical, chemical, and physicochemical methods

To obtain maximum PHA yield, ultrasonication, grinding biomass with Pestle and mortar, and glass beads vortexing of Bacillus cells with or without lysis buffer were studied. The yield obtained by these methods was compared with the standard method of recovery using 4% sodium hypochlorite followed by dissolution with chloroform. The hypochlorite resulted in up to 38% for 1 g/L biomass cell dry weight (CDW). The NPCM is differentially digested by sodium hypochlorite, resulting in higher purity. Nevertheless, as reported in the literature, PHB is significantly degraded by sodium hypochlorite, resulting in a 50% decrease in molecular weight. The chloroform was used to dissolve PHB, as mentioned in the literature. Chloroform can reduce the loss of PHA when used along with hypochlorite as a dispersion method (Hahn et al. 1994; Jacquel et al. 2008; Palmeiro-Sánchez et al. 2022). These solvents are cost-intensive and environmentally hazardous when used on a large scale (Jacquel et al. 2008; Bhat et al. 2023). Hence, the present study focuses on replacing these solvents with other sustainable extraction methods.

**Fig. 4:**
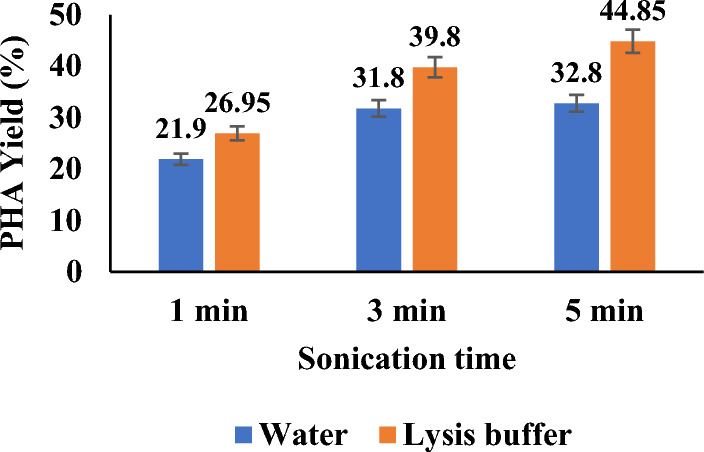
PHA yield (%) obtained by ultrasonication of *Bacillus* cells at varied conditions with and without lysis buffer

**Fig. 5:**
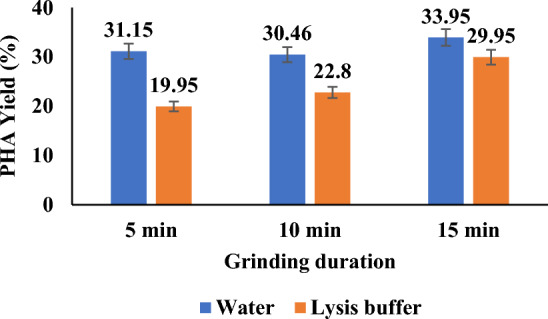
PHA yield (%) obtained by grinding *Bacillus* cells at varied conditions with and without lysis buffer using a Pestle and mortar

PHA obtained by ultrasonication and Pestle and mortar, both with and without lysis buffer, is shown in Figs. 4 and 5, respectively. As shown in Fig. 4, sonicating cells at the varied times of 1, 3, and 5 min, for 30 s on and off pulses, showed a gradual increase in PHA yield irrespective of the presence or absence of lysis buffer.

However, the presence of lysis buffer showed a slightly higher yield than in its absence within the same time intervals of sonication.

The literature supports that due to strong force and other factors such as prolonged time duration, there is slow degradation of the polymer chain (Hwang et al. 2006; Ishak et al. 2016). In the current study, it was observed that there was not much inclination in PHA yield when time was increased from 3 to 5 min with water. However, lysis buffer assisting sonication showed a sharp increase in PHA yield up to 45% in 5 min. Sonication tried above 5 min (result not in the graph) showed almost constant or lesser yield after that. The literature also supports such a pattern of PHA increase. PHA is intracellular, and in this study, the cells employed in PHA production is *Bacillus* sp, a Gram-positive isolate with a thick peptidoglycan layer, making it a tedious task for lysis (Divyashree and Shamala 2010).

Consequently, the lysis buffer with PMSF is designed to target this layer specifically. Therefore, efficiently assists in better lysis and sonication (Ramanan et al. 2008). The literature reports sonication in combination with other extraction methods. Sonicating the *C. necator* cells with sodium dodecyl sulfate (SDS) solution has been studied to release PHA (Arikawa et al. 2017). The results recovered PHA with a higher purity of 98%. Notably, the purity and production of PHA depend on the microbial strain, carbon source, and recovery method. PHA production, recovery, and microbial characterization are all correlated iteratively. As scientists tailor production requirements according to microbial characteristics, they can figure out the required recovery techniques depending on the microorganism’s features (Urtuvia et al. 2022).

Similarly, microbial characterization data can direct genetic engineering efforts to improve PHA synthesis. Thus, for the effective and long-term production of PHAs, a comprehensive strategy considering all three factors is necessary (Koller 2020; Sehgal and Gupta 2020; de Melo et al. 2023; Katagi et al. 2023). A study by Costa et al. reported that the various recovery methods produced PHAs with varying percentages of monomers, resulting in polymers with distinct monomeric compositions, molecular masses, and degrees of crystallinity. As a result, the properties of the polymers differed, which can impact their industrial application. The findings also showed that the chemical composition of PHAs synthesized by microorganisms is influenced by the carbon source used during PHA accumulation and the polymer extraction method (Costa et al. 2018).

For the first time, the current study combined sonication with lysis buffer and showed a maximum yield of 45%, whereas the absence of lysis buffer showed a 33% maximum yield. The trend observed can be relatively compared with a recent study of extracting PHA with a green solvent, such as natural deep eutectic solvent (NDES) in combination with sonication (Mondal et al. 2023), and was compared with results obtained with chloroform. Sonication of biomass, particularly when extracted with natural deep eutectic solvents (NADES), was crucial for efficient PHA release. In contrast, chloroform extraction showed minimal differences, likely because chloroform, as a chlorinated polar solvent, effectively disrupts cell walls for PHA release. In the case of NADES, ultrasonic waves play a key role in consistently releasing PHA by disrupting cell walls. However, as sonication amplitude increases, the process becomes more energy-intensive and physically disruptive (Mondal et al. 2023). Similar observations and results were claimed in studies that used sonication in cell disruption in combination with other extraction methods to disrupt cells and release PHA (Hwang et al. 2006; Rofeal et al. 2023; Zou et al. 2023).

**Fig. 6:**
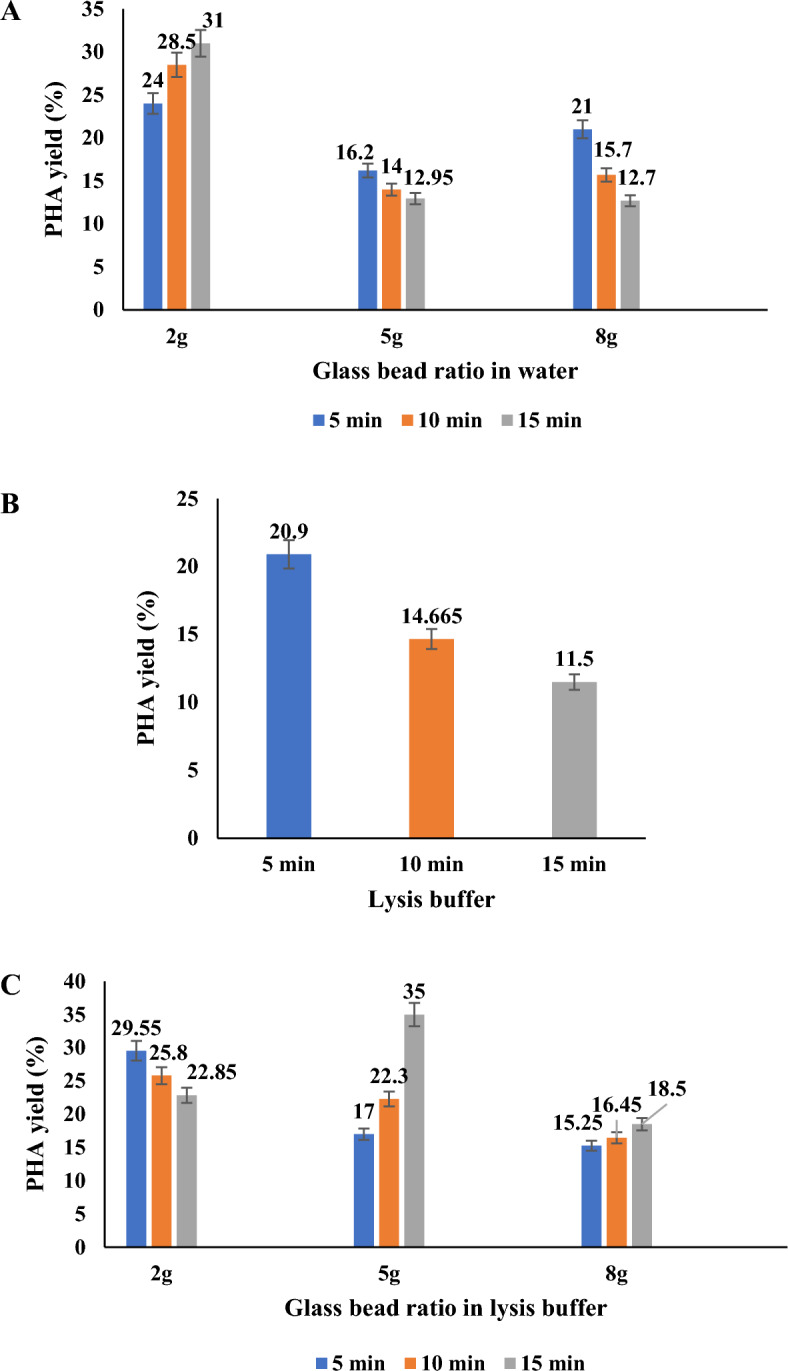
Physicochemical effect of *Bacillus* cell lysis and PHA recovery: **A** effect of glass beads. **B** Effect of lysis buffer. **C** Effect of glass beads in combination with lysis buffer

An approach using Pestle and mortar showed a maximum yield of up to 34% without lysis buffer. With the increase in time duration, little difference was observed in the yield for lysis buffer absence. The presence of lysis buffer showed a minimum yield in the 20–30% range without further increase, as shown in Fig. 6. This observation indicates that the abrasive force applied to the biomass during grinding may lead to the degradation of the polymer and cellular material. The presence of lysis buffer showed a further reduction in yield due to a greater degree of polymer chain degradation. Extraction of PHA by bead milling homogenization is the most common mechanical method due to successful scale-up possibilities and PHA recovery of 98% (Palmieri et al. 2021; Haque et al. 2022). From the thorough the literature review, the present study proposes cell disruption and PHA release by Pestle and mortar for the first time as a mechanical recovery approach.

In addition, the experimentation was performed to study the effect of extraction type on polymer characteristics, which was confirmed by polymer characterization. However, due to manual grinding force, this extraction method varies in final PHA yield. However, the grinding force can be controlled and show possible scale when similar parameters are applied to bead mill, homogenizers, grinders, with few optimization processes.

Glass bead vortexing was carried out with Bacillus cells as a physical force. The effect of glass beads, lysis buffer, and their combination for cell lysis of *Bacillus* was studied for maximum PHB yield. When biomass is vortexed with 2 g glass beads in the presence of water, it shows 31% PHB. The yield was increased with minimum glass bead ratio and maximum duration of vortexing. Meanwhile, overall yield decreased considering the increased bead ratio, as observed in Fig. 6A. A 10 mL lysis buffer, 5 min vortexing, and devoid of glass beads showed a maximum PHB of 20.9%, as in Fig. 6B. The study reported a maximum PHB yield of 35% when biomass was vortexed with 5 g glass beads and 10 mL lysis buffer for 15 min, as shown in Fig. 4C. The combination of glass beads and lysis buffer showed a sudden decline in yield with maximum bead ratio, as shown in Fig. 6C. The abrasive force of glass beads and the action of lysis buffer was the principle applicable in the effective cell lysis of *Bacillus.* In the increased ratio, in the absence of lysis buffer, the abrasive force exhibited on biomass by vortexing glass beads showed a decline in PHA release. Such an observation might be due to excess abrasive stress on biomass leading to cell lysis and degradation of released PHA. With 2 g glass beads and a maximum vortexing time of 15 min, it resulted in maximum PHA yield, hence being optimum condition. Vortexing biomass only with lysis buffer resulted in declined PHA yield in increased time.

The PMSF present in lysis buffer is well known to have protein-degrading properties. Hence, prolonged duration of its exposure to biomass disrupts cells as well as degrades polymer. Recent review reports that bead beating is a widely used technique for cell lysis to extract intracellular products like enzymes, DNA, and biopolymers. This method employs glass beads as cell-wall-disrupting agents, generating solid shear forces in a non-specific manner. An advantage of this approach is its applicability even at low biomass concentrations, ensuring the production of high-purity biopolymers (Haque et al. 2022).

It was not until 1923 that Maurice Lemoigne noticed that *Bacillus megaterium* could produce PHB. Several *Bacillus* species were employed to develop extraction techniques (Mohapatra et al. 2017; Palmeiro-Sánchez et al. 2022). Numerous production benefits are associated with *Bacillus* sp. These include the capacity to utilize a wide range of substrates, rapid growth, and high biomass production, faster PHA accumulation on nutrient-depleted conditions, the ability to produce a wide range of PHA copolymers (using different substrates and extraction methods), and robustness and tolerance to environmental conditions (Bhat et al. 2023; Vicente et al. 2023). Considering all these benefits, *Bacillus* sp was selected as a potential PHA producer in the current investigation.

Glass beads and lysis buffer were employed in cell lysis of *E.coli* cells to release total protein and interferon-α2b (Ramanan et al. 2008) as mechanical pre-treatment glass beads were used in PHA recovery from the mixed microbial culture with sonication (Samorì et al. 2015). For the first time, the current study investigated PHA recovery using sonication, Pestle and mortar, and glass beads with or without lysis buffer by modifying conditions from the literature for maximum PHA release.

The solvent extraction method in PHA recovery is the most common. However, it is tedious to handle hazardous chemicals on an industrial scale. The PHB extracted in the current study uses mild chemicals, following a sustainable recovery approach. Further studies can be done by varying the size of glass beads and other lysis buffers with varied concentrations. The lysis buffer in this experiment is known to alter the pH and break down the Bacillus cell wall and membrane, allowing access to intracellular PHA. The literature is available on applying different lysis buffers in releasing intracellular biomolecules, specifically bacterial cells (Islam et al. 2017). In the present study, the lysis buffer containing Tris, phenylmethyl sulfonyl fluoride (PMSF) (20 mM Tris–HCl, pH 8.0, 1 mM PMSF) was chosen because PMSF is well known for comprehensive cell disruption. The released PHA is prevented from degradation due to the conjunctive action of Tris–HCl with PMSF (Ramanan et al. 2008; Silva et al. 2018). Hence, lysis buffer and physical methods in combination provide efficient cell lysis and release of PHA without its degradation in the current study. Therefore, the physicochemical method of cell lysis reported in the current report provides scope for an economic and sustainable future.

### Characterization of polymer

**Fig. 7:**
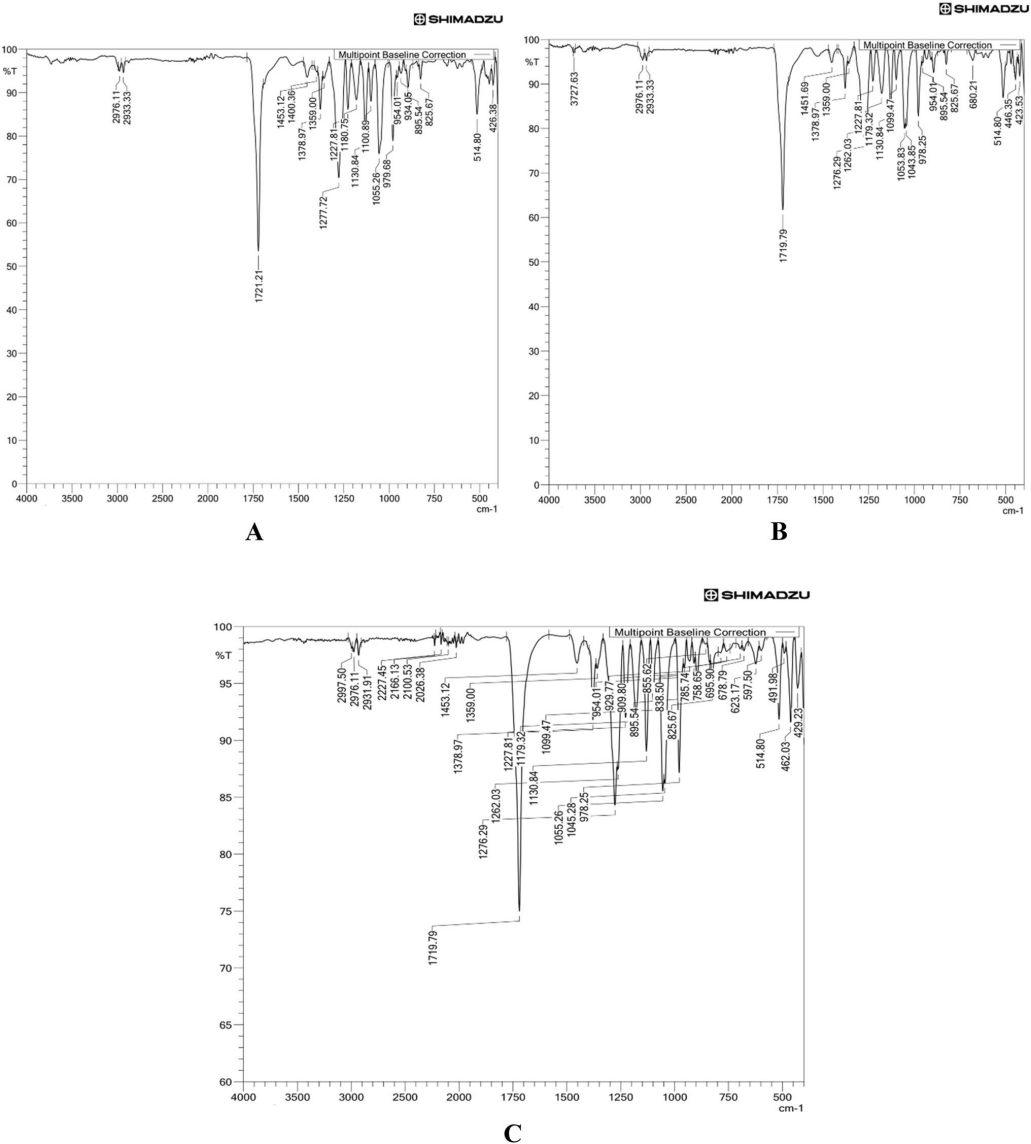
FTIR spectrum of **A** PHA extraction by Pestle and mortar. **B** PHA extraction by sonication. **C** Glass bead vortexing

FTIR and 1HNMR performed functional group and chemical structure elucidation of the extracted polymer. FTIR spectra shown in Fig. 7A–C showed sharp peaks between 1719 and 1721 cm^−1^ or polymers of sonication, Pestle, and mortar. This peak corresponds to C=O, a simple carbonyl compound, representing a characteristic homopolymer PHB band (Shamala et al. 2009; Morya et al. 2021).

**Fig. 8:**
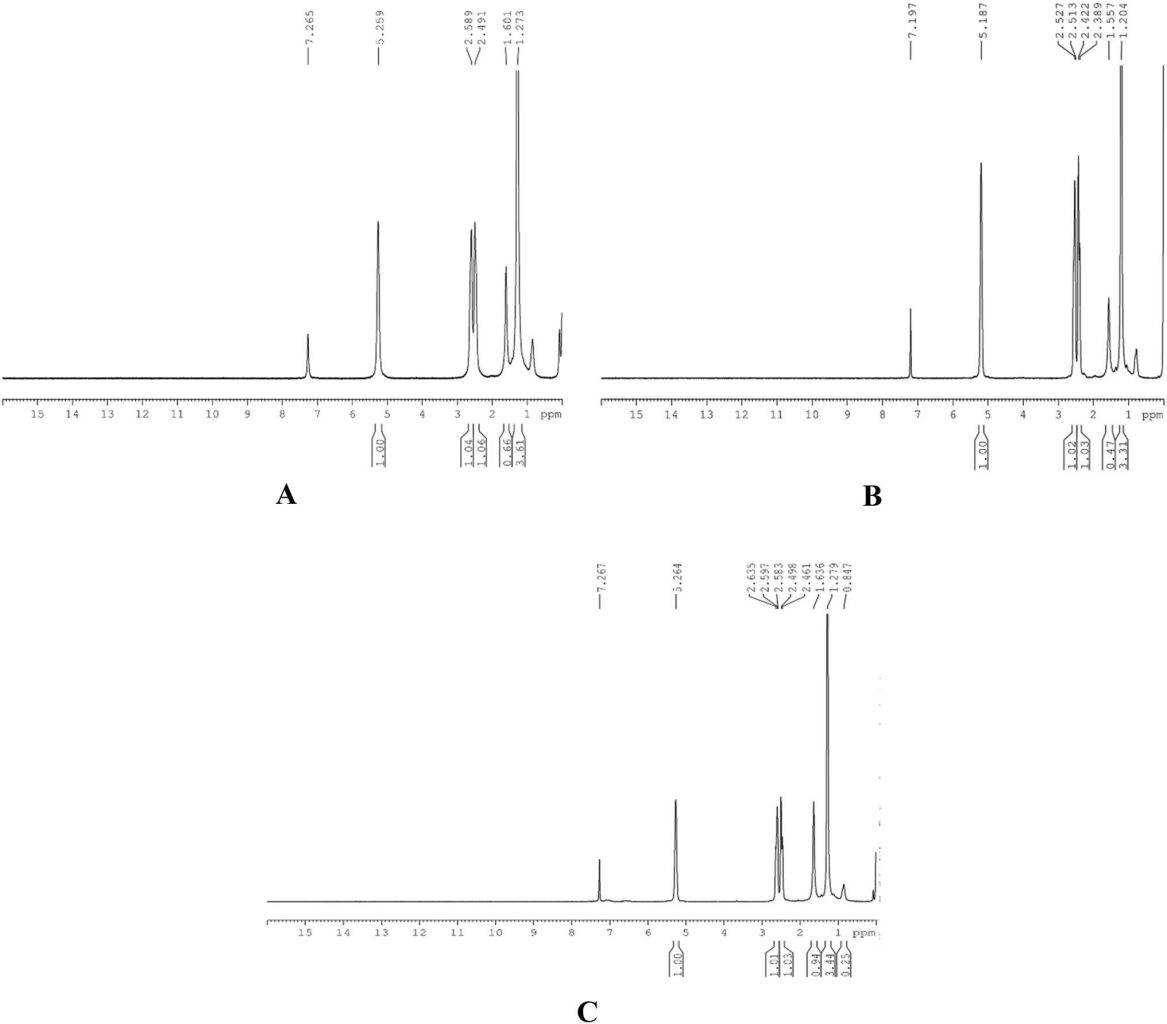
^1^H NMR spectrum of **A** PHA extraction by Pestle and mortar. **B** PHA extraction by sonication. **C** Glass bead vortexing

Absorption bands at 2976, 2931, 1276 (CH_3_ bend), 1045, 1055, 1099, 1130 cm^−1^ (C–O), 978, and 514 cm^−1^ were observed as characteristic bands of scl-PHA in the literature study (Shamala et al. 2009; Nandiyanto et al. 2019). Figure 8A–C shows ^1^HNMR peaks for polymers recovered by Pestle and mortar, sonication, and glass bead vortexing, respectively. ^1^HNMR peaks observed were 5.26, 2.46, 2.49, 2.58, 2.59, and 1.27, as shown in Fig. 8. According to the literature, these are the resonance expected for PHB (Sathiyanarayanan et al. 2013).

Signals at 1.10–1.62 and 0.8–0.9 ppm represent protons for methyl group. 2.35–2.72 and 1.75, 1.60 ppm as methylene protons and 5.2 signal for methylene. This signal range for protons reference was taken from the literature (Divyashree et al. 2009; Morya et al. 2021). Confirmation of PHB was done by gas chromatography. Data from G.C. also confirm that the bacteria produced PHB as a polymer from various extraction methods by *Bacillus* sp. The retention time (R.T.) was 4.35, 4.35, and 4.20 min for sonication, Pestle, mortar, and glass bead extraction methods. These R.T.s confirm the peak to be PHB. The R.T. was confirmed with the standard PHB that showed R.T. at 4.33 min.

## Conclusion

Recovery of PHB was studied by physical (sonication, glass bead vortexing, and Pestle and mortar), chemical (lysis buffer), and a combination of both, hence called the “physicochemical method.” Maximum PHA of 45% recovered from sonication in combination with lysis buffer that was due to conjunctive action of sonication and Tris–HCl-PMSF. Glass bead vortexing (5 g, 15 min) of *Bacillus* cells also showed improved yield of 35% in the presence of lysis buffer. Glass beads show a sustainable role as it can be reused for extraction. Both methods show scale-up possibilities. Grinding the cells (15 min) with Pestle and mortar, a laboratory extraction method, resulted in 34% of the maximum yield without lysis buffer. This approach shows uncertainty in yield due to the application of manual force. However, it is noteworthy that there is a significant difference in polymer recovery by Pestle and mortar, with and without lysis buffer. This method performs better without a lysis buffer, hence focusing on a cost-cutting strategy. The solvent extraction method in PHA recovery is the most common.

Nevertheless, it is tedious to handle hazardous chemicals on an industrial scale. PHB extracted in the current study uses mild chemicals, following a sustainable recovery approach. Further studies can be done by combining other methods with those discussed for maximum PHA recovery and an economical and sustainable future.

## Data Availability

The authors declare that the data supporting the findings of this study are available within the paper. any raw data files needed are available from the corresponding author upon reasonable request.
